# Microplastics as an Emerging Potential Threat: Toxicity, Life Cycle Assessment, and Management

**DOI:** 10.3390/toxics12120909

**Published:** 2024-12-14

**Authors:** Sameh S. Ali, Mohammed Hussein M. Alsharbaty, Rania Al-Tohamy, Maha A. Khalil, Michael Schagerl, Majid Al-Zahrani, Jianzhong Sun

**Affiliations:** 1 Biofuels Institute, School of the Environment and Safety Engineering, Jiangsu University, Zhenjiang 212013, China; rony.altohamy@ujs.edu.cn; 2 Botany Department, Faculty of Science, Tanta University, Tanta 31527, Egypt; mahakhalil9@yahoo.com; 3 Branch of Prosthodontics, College of Dentistry, University of Al-Ameed, Karbala 56001, Iraq; hussseinalsharbaty1986@gmail.com; 4 Department of Functional and Evolutionary Ecology, University of Vienna, Djerassiplatz 1, A-1030 Vienna, Austria; 5 Biological Sciences Department, College of Science and Art at Rabigh, King Abdulaziz University, Rabigh 25732, Saudi Arabia; malzhrani4@kau.edu.sa

**Keywords:** ecotoxicity, emerging contaminants, life cycle assessment, microplastics, socio-economy, waste management

## Abstract

The pervasiveness of microplastics (MPs) in terrestrial and aquatic ecosystems has become a significant environmental concern in recent years. Because of their slow rate of disposal, MPs are ubiquitous in the environment. As a consequence of indiscriminate use, landfill deposits, and inadequate recycling methods, MP production and environmental accumulation are expanding at an alarming rate, resulting in a range of economic, social, and environmental repercussions. Aquatic organisms, including fish and various crustaceans, consume MPs, which are ultimately consumed by humans at the tertiary level of the food chain. Blocking the digestive tracts, disrupting digestive behavior, and ultimately reducing the reproductive growth of entire living organisms are all consequences of this phenomenon. In order to assess the potential environmental impacts and the resources required for the life of a plastic product, the importance of life cycle assessment (LCA) and circularity is underscored. MPs-related ecosystem degradation has not yet been adequately incorporated into LCA, a tool for evaluating the environmental performance of product and technology life cycles. It is a technique that is designed to quantify the environmental effects of a product from its inception to its demise, and it is frequently employed in the context of plastics. The control of MPs is necessary due to the growing concern that MPs pose as a newly emergent potential threat. This is due to the consequences of their use. This paper provides a critical analysis of the formation, distribution, and methods used for detecting MPs. The effects of MPs on ecosystems and human health are also discussed, which posed a great challenge to conduct an LCA related to MPs. The socio-economic impacts of MPs and their management are also discussed. This paper paves the way for understanding the ecotoxicological impacts of the emerging MP threat and their associated issues to LCA and limits the environmental impact of plastic.

## 1. Introduction

Plastic products have been pervasive in all aspects of the economy and human activity due to their convenience and lightweight characteristics [1,2]. Plastic pollution has become a significant concern on a global scale as it poses significant risks to the health of biological systems and ecosystems [3]. Approximately 8.3 billion tons of plastic have been manufactured worldwide since 1950 [4]. Plastic is a highly successful product material because it is long-lasting, versatile, and inexpensive [5]. Every year, millions of tons of plastic waste are produced as a result of single-use packaging [6]. Approximately fifty percent of this plastic is immediately discarded following its initial utilization [7]. In 2019, global plastic use exceeded 460 million tons, although only 55 million tons were effectively recycled [8]. More than three years have passed since the beginning of the pandemic caused by the coronavirus 2019 (COVID-19) that swept across the world [5]. As a consequence, COVID-19 has caused an unprecedented rise in the production and consumption of personal protective equipment (PPE) [5,9]. This includes the production of medical masks, gloves, and protective clothing, all of which are needed for the management of the disease and for maintaining personal safety in day-to-day living [10]. Table 1 depicts the environmental fates of PPE [11,12,13,14,15,16,17,18,19,20]. Approximately 76 million gloves, 89 million medical masks, and 1.6 million medical protective eyeglasses were required to meet the monthly demand for PPE prior to the commencement of COVID-19, as reported by the World Health Organization (WHO) [21]. As a result of the COVID-19 pandemic, there has been an increase in the public’s utilization of PPE, with a projected monthly consumption of 65 billion gloves and 129 billion masks around the globe [22]. More than 35% of waste plastic is discharged into aquatic ecosystems on an annual basis, where it accumulates in both freshwater and marine habitats [23]. Recycling, incineration, and landfill disposal are the key technologies that are utilized for the successful management of plastic waste [24]. On the other hand, these technologies are limited by a number of variables, including the possibility of contaminating the environment, the high cost of implementation, and the difficulties of future application, all of which constitute obstacles to the effective management of plastic waste [25].

There are numerous degradation mechanisms that cause plastic to fragment into microplastics (MPs), including hydrolysis and photo-decomposition [26]. The potential for damage of biodegradable plastics increases as they decompose into MPs. A wide range of organisms from various habitats, trophic levels, and nutrition mechanisms have been observed to be impacted by these fragmented particles [27,28]. With its irreversible presence and broad influence on both terrestrial and aquatic ecosystems, the problem of MPs’ contamination in the environment is becoming an increasing concern [29]. It was in 1972 that significant progress was made in the investigation into the ecotoxicity of MPs in aquatic ecosystems [30]. It has been demonstrated that biodegradable MPs have a remarkable capacity to accumulate cadmium and facilitate its transportation to the digestive system [31]. Furthermore, the presence of biodegradable MPs has the potential to impact the composition and functionality of microbial communities, which are crucial for the cycling of nutrients, the degradation of organic matter, and the overall health of aquatic ecosystems [32]. MPs have been discovered to be capable of being consumed by fish, which could result in intestinal blockage in smaller fish [30]. Additionally, the presence of MPs in aquatic ecosystems has an impact on the overall condition of the ecosystem, such as algae and phytoplankton [33]. As freshwater MPs are detected in drinking water and demonstrate a threat to public health, there has been an increasing focus on this issue. Volatile organic chemical emissions from MP photo-degradation are responsible for two additional environmental concerns: climate change and air pollution, in addition to ecotoxicity [34]. Both the equator and the poles, as well as the sea and the land, have been found to be affected by the pollution caused by MPs [35]. This pollution has been found to be pervasive. MPs can infiltrate a range of paths, including sewage sludge, irrigation, littering, air deposition, flooding, and plastic covering. They are found in a variety of terrestrial habitats, including soil, and they can infiltrate through these pathways. These environmental loads may be further aggravated by the incremental MP discharge that is the result of the current development of plastic material production and use [2,3]. The prevalent use of plastics and the increase in their consumption will undoubtedly lead to a continued increase in the prevalence of MPs and their detrimental effects in the coming decades. As a result, it is imperative to avoid the use of hyperbole; however, the adverse effects of MPs cannot be ignored and must be appropriately captured by life cycle assessment (LCA) studies.

LCA is a quantitative decision-support tool recognized for its ability to identify alternatives or solutions that may exert minimal environmental impact [36]. It aids in the assessment and enhancement of the environmental sustainability of products and technologies over their entire life cycle [27]. The goal of LCA is to quantify the pathway that leads from stress to injury to ecosystems. This pathway comprises ecotoxicological impact pathways that are connected with chemical releases throughout the product life cycle [37]. A rising body of research has highlighted the fact that MPs are a type of plastic pollution that has a significant potential to create adverse effects on the environment [38]. As a consequence of this, it is of the utmost importance to incorporate the impacts that show the harmfulness of MPs to ecosystems as an essential component of LCA.

LCA evaluations fail to thoroughly investigate the potential risks posed by MP particles that are swiftly infiltrating the environment. In addition, the significance of LCA in MPs research is steadily increasing. Despite the existence of approaches that can approximate damage to ecosystem services within LCA, there remains a clear deficiency. Therefore, it is urgent to address this gap by extending LCA to include MPs and their potential ecotoxicological impacts. To this end, this review expands upon previous reviews by exploring the various aspects of MPs, including their formation, distribution, and methods for their detection. This review emphasizes the effects of MPs on ecosystems and human health. It also assesses LCA related to MPs, the socio-economic impacts of MPs, and their management.

## 2. Methodology

**Figure 1:**
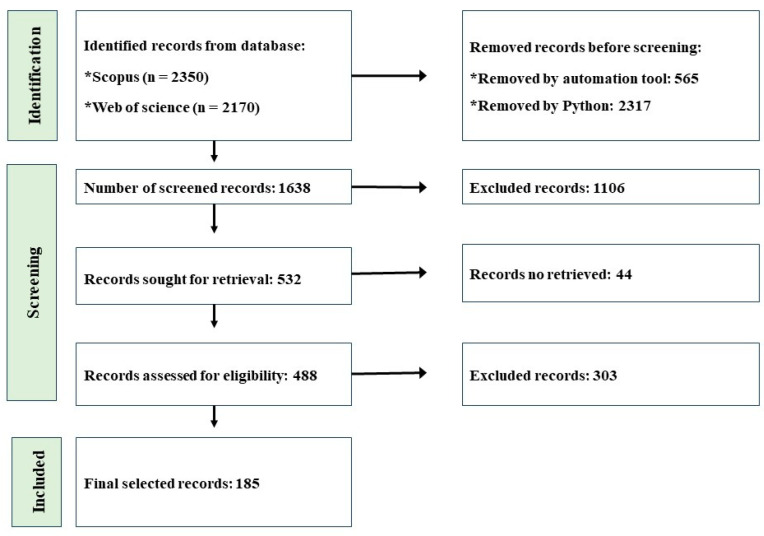
Flow diagram of the selected and identified studies from the databases.

A systematic approach was implemented to identify pertinent literature from databases in order to guarantee a comprehensive review. In this study, academic publications were sourced from the Web of Science and Scopus databases, employing the Preferred Reporting Items for Systematic Reviews and Meta-Analyses methodology [39]. The methodology for this review paper was restricted to articles written in English and encompassed data identification and extraction, data screening, and eligibility analysis, covering the period from 2014 to 2024 (Figure 1). Articles were selected based on a variety of criteria, such as the topic’s relevance, the publication date, and the presence of substantial reviews or experimental data on plastic waste ecotoxicity and LCA. Due to its relatively recent emergence, the term “microplastic” was not incorporated into the search phrase, which may result in the identification of a limited number of studies. The search was performed utilizing the following keywords: “plastic waste”, “life cycle assessment”, “plastic ecotoxicology”, “life cycle impact assessment”, “aquatic and terrestrial plastic”, “adverse impact of plastics on human”, “oral ingestion”, “life cycle inventory”, and “life cycle analysis”. The elimination of studies published before 2014, records in languages other than English, books, and book chapters was accomplished by the utilization of an automation tool, as depicted in Figure 1. As a result, a total of 565 publications were removed from consideration. The elimination of duplicates and articles that did not include the terms “plastic waste” or “life cycle assessment” in the title, abstract, or keywords was accomplished through the utilization of a Python script that was specifically designed for this purpose. Additionally, this removed more than 2300 records that were identical to one another. The remaining 1638 studies were preliminarily screened by scanning the title and keywords, as mentioned above. It is considered that the research corpus reflects the prevalent research tendencies in the LCA area for plastic waste items. In the first stage of the screening process, a total of 1106 research papers were disqualified from consideration. After making an effort to obtain the remaining 532 records, it was discovered that 44 of them were not accessible. The papers that were retrieved were subjected to a comprehensive evaluation for their eligibility. There were a total of 185 records that were included in this study.

## 3. Mechanism of Microplastic Formation

**Figure 2:**
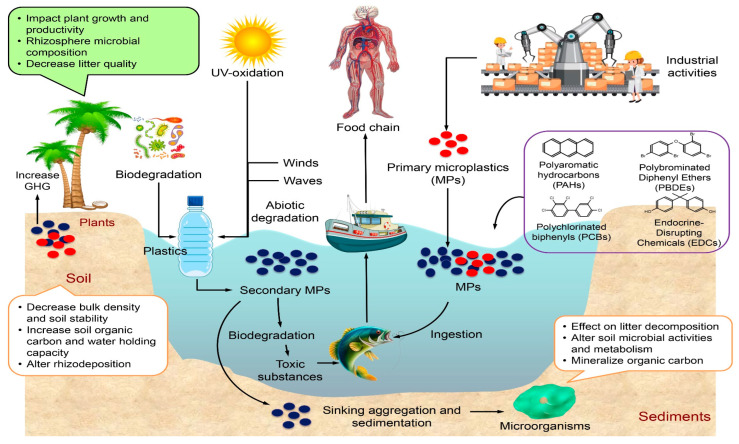
Formation of primary and secondary microplastics and adverse effects of their formation on aquatic flora and finally to human via food chain.

The fragmentation and degradation of massive plastic debris are the principal sources of MPs in aquatic environments (Figure 2). MPs have the potential to move through the atmosphere by means of wind or rain, reach the surface of the water from the land, and then proceed to enter the ecosystems of both freshwater and marine environments [40]. As soon as the MPs are introduced into the water, they will undergo a steady process of decomposition and breakdown due to the influence of ultraviolet radiation, biological habitat [41], and disturbances in the water (Figure 2). Despite the fact that the production of MPs from large plastic particles happens quite quickly, the process of breaking down MPs into smaller particles and then mineralizing them is a lengthy process [2,3]. Ultraviolet (UV) light is the principal catalyst that is responsible for the creation of MPs. As a result of their microscopic size, MPs are easily ingested by zooplankton, filter feeders, and benthic organisms in aquatic habitats. This might result in clogs within the digestive tracts of marine fauna [42,43,44,45]. Furthermore, the hazardous substances that are found in MPs have the potential to cause bioaccumulation and toxicity, in addition to causing disruptions to the digestive system [46]. Consumption of these persistent toxic pollutants by consumers can lead to their absorption and retention, which can have adverse effects on their health (Figure 2).

## 4. Distribution of Microplastics and Their Potential Risks to the Environment and Human Health

MPs can be present in all environments [1]. MPs are released through a variety of pathways, including human utilization and applications of plastics, industrial activities, and transportation, with the majority entering the marine ecosystem via direct disposal or indirectly via river transport (Figure 2).

### 4.1. Soil

Soil provides microorganisms with a habitat and the nutrients they need to survive, ensuring that they will continue to exist [47]. When it comes to soil, MPs perform the role of mediators, making it easier for bacteria, fungi, and other microorganisms to colonize and move from one phase to another. Researchers detected MPs at concentrations of 78 N/kg in shallow soils and 62.5 N/kg in deep soils [48]. Multiple factors influence the distribution and incidence of MPs in soil, including soil characteristics, soil biota, and agricultural activities [49]. MPs that are located on the surface of soil have the potential to be carried across a considerable distance as a result of the wind action [50]. Through tillage operations, MPs can be moved from the topsoil to the deeper topsoil. Through the process of leaching, which is a common physical process, MPs are frequently released into groundwater [51]. There is a substantial relationship between the shape of MPs and their transportation in soil. MPs that are spherical or granular are more likely to be carried to the deeper layers of the soil. However, the movement of MPs inside the soil may be restricted for a variety of reasons, including interactions with different types of films and fibers [52]. MPs have an effect on both the physical and chemical properties of soil. When 0.4% polyester microfiber is added to loamy soil, it has been observed that the water holding capacity and rate of evaporation of the soil may be dramatically enhanced, and the bulk density and water-stable soil aggregates can be significantly reduced [53]. Additionally, considerable concentrations of polypropylene MPs (greater than 28%) were found to dramatically enhance the amounts of phosphorus, nitrogen, and organic carbon in soil, as well as promote the release of soil nutrients [54]. Furthermore, the utilization of mulch plastic residues in the range of 67.5 to 337.5 kg/ha has the potential to decrease the organic content of the soil, which in turn might have an effect on the fertility of the soil [55].

MPs have a detrimental effect on a variety of soil organisms, such as earthworms, nematodes, and mites [56]. It is possible for MPs to bioaccumulate within food chains, which can have negative effects on organisms at several trophic levels [57]. It is possible that the migration of MPs into the soil ecosystem will have a negative impact on the environment. This is because the MPs may produce hazardous substances that have become adsorbed on the surface of the MPs [58]. Direct biotoxicity to soil microorganisms may be the result of oxidative stress brought on by reactive oxygen species (ROS) and/or the toxic influence of MP additives that have leached into the soil [59]. Through the reduction in soil microbial activity and the modification of the plant nutrition cycle, MPs in the soil have the potential to exert an indirect influence on the germination of plant seeds and the growth of seedlings [60]. The change in soil pH that is caused by the presence of MPs has the potential to induce direct toxicity to soil microorganisms by reducing their enzyme activities and cell metabolism [61]. Alternatively, the change in the soil pH could produce indirect toxicity by simultaneously limiting the availability of nutrients and increasing ionic toxicity [62]. The primary focus of current research has been the potential impact of MPs on microbial activity, as determined by alterations in enzymatic activities. The impact of a variety of MPs has been investigated on the microbial activity of soil samples, including polyamide, polyethylene, polypropylene, and polyester [63]. The metabolism of microorganisms was significantly enhanced by polyethylene and polyamide. Nevertheless, the microbial metabolism was reduced by polyester and polypropylene [63]. Soil organisms, such as earthworms, have the ability to actively contribute to the movement of MPs through the processes of ingestion and excretion [64]. The imbalance in the availability of nutrients in soil, which is induced by the adsorption of nutrients by MPs and the increased demand for nitrogen by soil microbiota, may possibly result in changes to the organization of the microbial community as well as their associated functions [65]. The introduction of low-density polyethylene MPs into dry soil at a concentration ranging from 0.2 to 1.2% (*w*/*w*) was found to be detrimental to earthworms and ultimately resulted in their death [66]. Earthworm growth was significantly decreased at MP concentrations ranging from 1% to 2% (*w*/*w*), which resulted in effects that were sufficient to cause death [67]. The adverse impacts of MPs on soil microbial enzyme activities are given in Table 2 [68,69,70,71].

### 4.2. Aquatic Environment

The ocean is the principal habitat of MPs, and plastic trash comprises approximately 80–85% of marine litter [72]. The global plastic manufacturing industry has improved over the past 60 years, resulting in an increase in the number of MPs in the oceans. These MPs reach the marine environment through a variety of channels [73]. Plastic fiber fragments from clothing and MPs in cosmetics can infiltrate the water through domestic or industrial drainage systems [74]. In Europe, the Danube River releases 4.2 tons of plastics into the Black Sea each day, and the Rhine River contributes around 25.5 tons of MPs annually to the North Sea, affecting Belgium, Denmark, France, Germany, Great Britain, the Netherlands, and Norway [75].

Plastic additives have the potential to disrupt the environment by discharging potentially hazardous chemicals and posing a significant threat to the biota, even in small quantities [76]. The primary environmental implications of MPs are the potential injury to all life forms, the negative effects of additives, and the chemical effect of combined pollutants that adsorbed on MPs’ surface. It is postulated that MPs with a high specific surface area are more resistant to additive absorption [77]. Consequently, the consumption of MPs by aquatic biota can result in endocrine disruption and potentially affect locomotion, development, and reproduction [78]. MPs ingested by bivalves and corals may persist within the organisms and traverse their tissues [79]. Due to the surface hydrophobicity and area of MPs, they can adsorb dangerous contaminants from their environment. MPs can amalgamate with veterinary antibiotics in consumable bivalve species [80]. Triclosan-adsorbed MPs restricted microalgae development more than individual MPs due to their combined toxicity, which can cause inflammation and immunological harm, finally resulting in organism mortality [81]. The presence of MPs greatly exacerbates the immunological toxic effects to two veterinary antibiotics (oxytetracycline and florfenicol) in a bivalve species [82]. Exposure to these antibiotics led to a significant alteration in hematic parameters, inducing the production of ROS. This exposure also led to the reduction in serum lectin content, DNA damage, and suppressing the viability of hemocyte [82]. MPs and poly aromatic hydrocarbon combinations exert toxic impacts on the hematic parameter in a marine bivalve species *Tegillarca granosa* (Linnaeus, 1758) [83]. This combination led to changed hematic composition, decreased total hemocyte count, and inhibited the phagocytosis of hemocytes [83]. The ecotoxicity of MPs on aquatic organisms is summarized in Table 3 [84,85,86,87,88,89,90,91].

### 4.3. Air

Synthetic textiles are the principal source of airborne MPs [92]. Synthetic fibers are widely employed worldwide due to their exceptional abrasion resistance and tenacity, as well as their pleasant feel and contact. The washing, drying, and wearing of clothing and other fiber commodities results in the release of minute fibers. A single laundry is purported to liberate approximately 1900 fibers [93]. Moreover, the cutting, chopping, and grinding of synthetic fabrics result in the production of vast quantities of minute fibers. In addition to synthetic textiles, the environment contains a plethora of sources of MPs. Possible sources of MPs in the atmosphere include synthetic particulates in dried sewage sludge, landfilling sites, mulching plastic films, plastic-containing soils, and waste incineration sites [94]. MPs in the atmosphere may also originate from synthetic fibers included in sludge from wastewater treatment facilities, particulates, and microbeads [95]. Furthermore, intense traffic generated a significant amount of airborne MPs. MPs were emitted into the atmosphere as a result of tire abrasion and the suspension of dust on the road caused by wind and vehicular traffic flow [96]. Airborne MPs are prone to contaminating land and aquatic environments. Airborne MPs can travel considerable distances and accumulate in the marine environment due to wind currents. Conversely, the extent to which aqueous MPs contribute to airborne MPs remains ambiguous [97]. Airborne MPs may be deposited on terrestrial surfaces and subsequently transmitted into aquatic environments through surface runoff and rainfall or re-suspended into the atmosphere by wind [97]. Moreover, snow and rain were considered to be the principal contributors to MP deposition in remote locations, attributed to the transfer of MPs among aquatic, terrestrial, and atmospheric ecosystems, creating a dynamic cycle of MPs within the environment. MPs may be transported downward through runoff into groundwater or soil as snow evaporates, thereby influencing the physicochemical properties of soil and groundwater. MP fluxes from snow and rain events in Gdynia were substantially greater than those from rainfall events of comparable intensity [94,96].

### 4.4. Human Health

**Figure 3:**
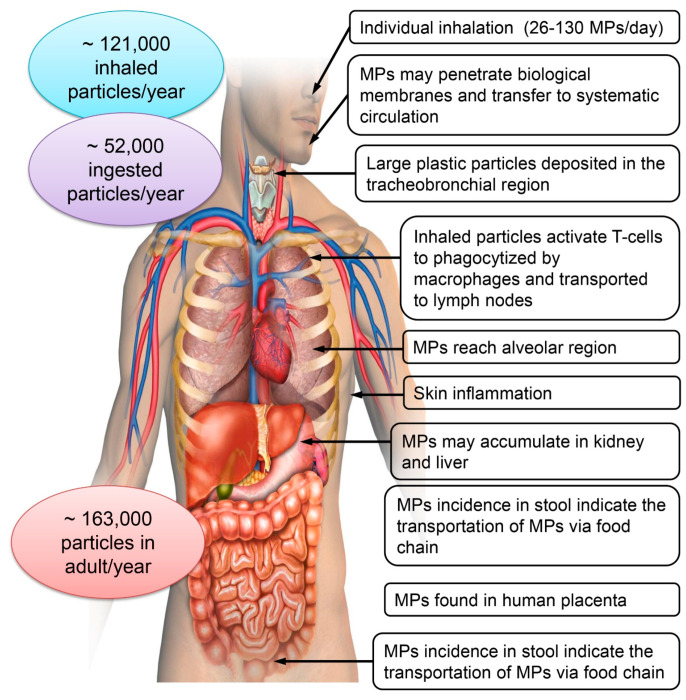
Impact of microplastics on human health.

The extensive exposure of individuals to MPs via the inhalation of airborne particles and the ingestion of particles included in dust, water, and food is well recognized (Figure 3). To contextualize, it is believed that humans consume tens of thousands to millions of MP particles annually, amounting to several milligrams daily [98]. Atmospheric MPs may traverse the respiratory system and accumulate in the pulmonary tissues of both children and adults [99]. Inhaled MP particles can penetrate the human body through the respiratory system and access the alveoli. Larger particles are obstructed by mucociliary clearance in the upper respiratory tract, which encompasses the nostrils, nasal cavity, mouth, pharynx, and larynx. MPs can circumvent and persist in the pulmonary depths for an extended duration [100]. Human exposure to marine MPs occurs through ingestion and inhalation (Figure 3). Based on age and sex, the estimated annual intake of MPs by certain humans is between 39,000 and 52,000 particles [101]. MPs increase to between 74,000 and 121,000 particles when inhalation from the air is taken into account [102]. The use of atmospheric conveyance would allow for members of parliament to travel up to 95 km and explore more isolated places [103]. Humans have the potential to consume MPs in a variety of different ways, including the consumption of edible fruits and vegetables. Conti et al. [104] found that apples had the highest amount of contamination, with a median of 223,000 p/g of MPs. There is a higher frequency of MPs in aquatic species, according to a study that was carried out in China [105]. This implies that the health risks connected with commercial fish and bivalves that are sold in urban markets are greater than those that are associated with fish and bivalves exported from other countries.

As a result of the molecular pathways that interact with MP particles, oxidative stress, inflammatory reactions, and metabolic problems may be detrimental to health [106]. These effects may be deleterious to health. According to toxicological tests conducted by Deng et al. [107], it has been discovered that MPs, which have a diameter ranging from 5 to 20 µm, tend to collect in the liver and kidneys of rodents. This accumulation leads to inflammation and alterations in their metabolic profiles. MPs with a size of less than 20 µm were observed to be capable of traversing biological membranes [108]. Despite the fact that plastics were once thought of as harmless materials, there is evidence that exposure to MPs in experimental animals is linked to a number of diseases. These disorders include inflammation, immunological response, disturbance of the endocrine system, and changes in the metabolism of lipids and energy [2,3]. It is important to be concerned about the possibility of being exposed to MPs; nevertheless, it is also possible for MPs to be sources of exposure to plastic additives and other toxicants. Inhaled MP particles were shown to activate T-cells, which led to the transfer of particles to lymph nodes and an elevated risk of cancer [109]. Additionally, MPs have been demonstrated to induce tissue inflammation and have toxic effects on the human nervous system and liver [110]. Infertility, immunotoxicity, metabolic alterations, and changes in olfactory behaviors are among the other adverse effects [111]. Although there is currently insufficient definitive evidence to establish a direct correlation between MP consumption and human health, correlative studies in individuals exposed to high concentrations of MPs, as well as model animal and cell culture experiments, indicate that MPs may induce reproductive and developmental toxicity and elicit immune and stress responses [102].

Dentists and other dental professionals are susceptible to inhaling MPs when they work in dental establishments [112]. MPs are discharged during routine dental procedures, which may pose a possible threat to the health of the patient. Many different types of polymers are utilized in the dental industry for the purposes of packaging and treatment [113]. For the purpose of determining the potential health hazards that dental professionals face as a result of inhaling and ingesting MPs, it is vital to monitor and evaluate the polymer hazard index and MPs in dental units [112]. During dental operations, dust comprising MP particles from ceramics, metals, and acrylics can be generated through grinding, polishing, and sandblasting [114]. The surface of MPs can establish carbonyl bonds, leading to the adsorption of pollutants like heavy metals, contingent upon their composition. The possibility of increased pollution underscores the need for more investigation and preventive strategies [115].

Additives not only impart essential characteristics of color and clarity but also confer resistance to degradation from the ozone and temperature fluctuations and provide thermal/electrical resistance [116]. Numerous compounds have been recognized as endocrine disruptors, including bisphenol A, phthalates, and some brominated flame retardants. These compounds have been identified as detrimental to human health [117]. As resin composites have emerged as a viable alternative to dental amalgam, the presence of these chemicals in dental restorative composites is a matter for substantial worry in the area of dentistry [118]. This is because plastic composites have become increasingly popular. Composites have a greater number of advantages than amalgam due to their visual appeal and the ideal restoration methods that they present. Because of these characteristics, which typically outweigh the risks that are present in the area, they are frequently favored [119].

There is a substantial level of uncertainty among practitioners, and the number of studies on polymer exposure from dental materials is extremely limited [120]. Despite the high volume of cross-sectional studies that are conducted to evaluate the knowledge of practitioners regarding hazardous and general refuse, it would be advantageous to conduct a similar study on an additional potential source of toxicity, such as polymer exposure from dental materials. The mission of the research that is now being conducted in the field of dentistry is to investigate the use of MPs, the levels of toxicity that are present in various composite resins, and the effects that these resins have on both human and environmental health [121]. The application of MPs on a daily basis in a variety of routine medical, surgical, and diagnostic procedures is connected with the increased risk of exposure to these particles [122]. MP fibers may be produced by certain PPE and surgical materials that are utilized by medical personnel during surgical procedures. These materials and equipment include surgical gloves, gowns, and sutures [123]. The use of orthodontic appliances (elastics and aligners) and preventative treatment (mouthguards and nightguards) might result in the production of MP fragments [124]. Oral hygiene products like toothpaste, mouthwash, and polishing pastes are responsible for the release of MP particles during routine dental scaling and cleaning operations [125]. As shown in Table 4, the toxicological consequences of various types and shapes of MPs are now being taken into consideration [126,127,128,129,130,131,132].

## 5. Microplastic Detection Approaches

The utilization of analytical methodologies is necessary for the quantification and characterization of MPs. The major technique for MP detection now involves the study of chemical and morphological properties [133]. The selection of detection and analytical methods is influenced by the characteristics that distinguish MPs, such as their small size and the presence of several additives [134]. Raman spectroscopy and Fourier transform infrared (FTIR) spectroscopy are the two microscopic–spectroscopic techniques that are utilized the most commonly for the identification of MPs [135]. The eradication of both organic and inorganic matrix components is often required for these techniques, which necessitates the completion of a complex sample preparation process [136]. The determination of the mass content of MPs in environmental samples is typically accomplished through the utilization of thermoanalytical techniques, such as thermal extraction desorption–gas chromatography/mass spectrometry (TED-GC/MS) [137], pyrolysis-GC/MS (Pyr-GC/MS) [32], and differential scanning calorimetry [138]. The determination of the mass content of MPs is also accomplished through the utilization of chemical techniques, such as inductively coupled plasma mass spectrometry [139] and chemical extraction combined with liquid chromatography with UV detection [140]. The infrared spectroscopy, Raman spectroscopy, and thermal analysis techniques, on the other hand, are not ideal for the complete monitoring of MPs because they are only able to measure a restricted number of polymers that are relevant to the environment [141]. The methods used for the detection of MPs are given in Table 5 [142,143,144,145,146,147].

## 6. Microplastic Life Cycle Assessment and Management

Life cycle assessment (LCA) was first implemented in the plastics industry in the late 1960s, making it one of the earliest adopters of this methodology [148]. An improved understanding of the environmental impact of the plastics sector can be achieved through the utilization of LCA, which involves the examination of five distinct phases of the life cycle: material extraction (cradle), manufacture (gate), usage (customer), distribution/transportation, and end-of-life (grave) [149]. It is possible for research to choose to examine all phases of the value chain (for example, cradle-to-grave), or it may choose to concentrate on particular components of the value chain (for example, gate-to-gate). LCA has achieved significant success in optimizing process design, justifying policy implementations, evaluating trade-offs among available options, and assessing the most appropriate materials for a particular application since its introduction to the plastic sector [150].

LCA is one of the methods that has been utilized by a number of researchers in order to take into account the effects that MPs have on both human and environmental health [151,152]. LCA is a process that is based on scientific principles and assesses the environmental consequences of products across their whole life cycle. This includes the extraction of raw materials, manufacture, transportation, consumption, and disposal of the product [153]. LCA is a globally recognized methodology that is currently employed as a means of facilitating policy-making [154]. Corporations and governmental bodies, such as the European Commission’s Joint Research Center, have adopted it after it transitioned from the academic domain. Consequently, it has served as an inspiration for numerous initiatives, including the LCA Initiative [155]. LCA is a technique that allows for the evaluation of the effects of a wide range of policies, such as the ban on single-use plastics in Europe, the Plastic Waste Control in South Korea, the impending ban on single-use plastic in Canada, and the prohibition of Styrofoam containers in restaurants in San Diego [156]. Recently, the Sustainable Development Goals (SDGs) of the United Nations have been adopted in the policy sector, which incorporates life cycle thinking. These goals aim to promote sustainable consumption and production practices [150,157].

The efficacy of the circular economy (CE) is evaluated through material flow analysis, end-of-life (EoL) treatment, and eco-efficient product design, employing life cycle evaluation [158]. Life cycle thinking is being evaluated to validate assertions concerning greenhouse gasses (GHGs) and to encourage sustainable buying practices. It seems reasonable to consider the application of LCA to examine potential problems related to plastics. With the growing concern regarding plastic and MP pollution, recycling has become an indispensable solution to this issue. However, in order to be recyclable, its refuse necessitates mechanical and chemical treatment [159]. As a result, an environmental assessment is necessary to assess the numerous recycling alternatives. The environmental consequences of plastic recycling have been examined, and the results indicate that the recycling of polyethylene terephthalate (PET), for example, has the potential to reduce carbon emissions by up to 30% and conserve energy [160]. In addition, increasing the recycling of the plastic used to package beans by 5% would lower the potential for global warming by 7% [161].

To date, there have been only a few publications that have attempted to develop numerical models and monitor the loss of plastic in the environment in order to provide a plastic footprint [162,163,164]. These are useful methods to derive total global quantities of plastic lost as macroplastic and MP. However, the preponderance of these methodologies, to the best of our knowledge, does not establish a connection between the final impact and the loss of plastic. Most of them fail to account for secondary MP formation and not for the decomposition changes (size changes and gas products) that mismanaged plastic will undergo throughout its EoL. By adopting the waste collection and treatment pathways that were in place at the time of this study for the same product and/or material category, it is recommended that realistic EoL scenarios be developed for goods that are not currently accessible for commercial use. After the product in issue has been released onto the market, these circumstances are potentially applicable to the product in question [165]. One can ascertain the proportions of distinct EoL alternatives by utilizing data pertinent to the same product category as the project’s focus, or, when applicable and relevant, to the specific existing product(s) that the product aims to rival [166].

A portion of the product in question may be discarded as debris in the environment at the EoL, resulting in the introduction of macroplastic and MP into the riverine and/or marine environment [167]. The intentional EoL option for (plastic) products is not littering; rather, it is a result of waste mismanagement practices and/or improper consumer behavior. Accidents, like littering, are typically not taken into account in LCA. However, in light of the current relevance of this environmental issue for plastic products, EoL scenarios should also consider the proportion of the product that is littered, as well as the corresponding burdens and impacts [168]. The establishment of a connection between the formation of secondary MPs and the rates of global loss is not only advantageous but also essential in order to ascertain the ultimate concentrations of plastics in a number of different environmental compartments. Consequently, it is conceivable to extract damage indicators, such as the loss of species as a consequence of MP consumption to name one example. There have been secondary MPs implemented for certain categories of plastic, and Ryberg et al. [169] have calculated the amount of plastic that is lost on a global scale. In their study, Kawecki and Nowack [170] approximated the amount of MP that was present by using the building sites and manufacturing data. In none of these investigations is there any evidence to suggest that there is a connection between the particles and an indicator of potential damage or ultimate impact.

Through the publication of standards 14040:2006 [171] and 14044:1997 [172], the International Organization for Standardization (ISO) has provided documentation of the particular needs for LCA. The four stages of an LCA are as follows: (i) the defining of the purpose and scope of the project; (ii) the life cycle inventory analysis (LCI); (iii) the life cycle impact assessment (LCIA); and (iv) the interpretation of the results. The purpose and breadth of an assessment are summarized by the definition of the goal and scope. Data acquisition and quantification of inputs and outputs are essential components of an LCI. In the context of specific environmental and human health impacts, an impact assessment evaluates the data collected during the LCI stage. Ultimately, the interpretation stage re-establishes the assessment’s objective by linking the results of the LCI and LCIA stages. These stages may be iteratively revised to progressively enhance the model as additional information is gathered [171,172]. A functional unit (FU) is a quantified performance of a product system that is used as the reference unit in an LCA study, as defined by the ISO standards [173]. To date, there are no LCAs that establish a connection between the production of particulate matter and the potential adverse effects it may have on human or environmental health. The endeavor to standardize inventory gathering, which might be related with a particular geographical region, is considered to be one of the factors that is lacking in the majority of plastic loss inventories. As a consequence of this, more inquiries are still being conducted. These inquiries include the relationships between the various practices and strata of waste treatment infrastructure that pertain to plastic losses, as well as the implications of different topographies on the redistribution, final fate, and environmental impact of MPs [174].

There is still a substantial dearth of conversation addressing MPs, despite the fact that LCA is utilized extensively in the plastics business. Even in a study that specifically investigated cosmetic products that contained microbeads made of plastic [175], the effects of MPs were only investigated in terms of production and distribution. As a result, the long-term toxicity risks to human health and ecosystems were not taken into consideration. We are of the opinion that there are numerous inherent restrictions that further impeded the incorporation of MPs into the LCA framework. This exclusion may easily be attributed to the considerable information gaps in MP research; nevertheless, we are of the opinion that there are several limitations that would be considered inherent.

### 6.1. Life Cycle Assessment Tools

The processes of the environment are frequently intricate and complex. However, this complicates the modeling process of an LCA. In addition, LCA is frequently data-intensive. As a result, the user is assisted in the administration and modification of these data volumes through the use of appropriate software tools and computers [176]. In addition to presenting and analyzing the results, the LCA program also illustrates the process chains, further organizing the modeled situation [177]. There is a wide range of applications for LCA software tools, regardless of where the LCA technique is in use. In accordance with ISO 14040 [171], the primary purpose of LCA is to assess the environmental characteristics and potential consequences that are linked with a product. In addition, environmental hot spots, which are defined as processes that have a significant influence on the environment, can be discovered. Consequently, it is possible to adopt a production method that is environmentally friendly in the site that is most effective. Moreover, LCA has the potential to make it easier to execute a production process that is more environmentally friendly [178]. It has the potential to enhance and optimize resource management, which will ultimately lead to a more effective utilization of both energy and materials. The fundamental objective of LCA is to assess a number of different alternatives and determine which one is the most ecologically friendly option [179]. Environmental LCA and LCA software serve as supportive instruments in the process of decision-making. Environmental LCA software tools are given in Table 6.

### 6.2. Socio-Economic Impacts of Plastics

The productivity and efficiency of commercial aquaculture and fisheries are reduced by the physical entanglement and damage caused by plastics that aquatic organisms ingest [180]. They have a direct effect on marine life, particularly fish populations, which in turn affects the entire food chain [181]. Seafood is the principal source of animal protein for 1.4 billion individuals, or 19% of the global population, and accounts for over 20% of their dietary consumption by weight [182].

Recreational activities have been directly affected by marine plastics [183]. In marine environments, plastic has been observed to float on the ocean’s surface and land on the shore, resulting in significant contamination along the littoral [184]. Consequently, visitors refrain from visiting beaches, resulting in grave socio-economic repercussions. It is imperative that the tourism sector of the nation expands, and resorts have to be established in coastal regions. Nevertheless, the recreational qualities of the shoreline are being diminished and social, psychological, and mental stability are compromised as a result of the degradation of the shoreline by plastic [185].

Disposable plastics with intricate chemical compositions are becoming more prevalent in the environment as plastic products are widely used. Cities struggle to manage waste socially and environmentally appropriately due to rising urbanization. Cultural, environmental, socio-economic, and institutional factors and institutional skills affect how locally generated waste is handled and how effective alternative solutions may be used [186]. Around the world, waste governance is becoming increasingly regional and standardized. In developed nations, where inhabitants produce much more waste than other residents, management is formally handled on a municipal or provincial level [187]. Waste management is officially dealt with at the municipal or regional level in industrialized nations, where residents produce far more waste than other residents. It is necessary to integrate the informal waste sector into the development of towns and cities, reduce consumption in developed cities, manage more complex waste, and increase and standardize solid waste data collection and analysis in parallel while protecting human health [2,3]. Waste management is seen differently in various nations. Furthermore, in 2010, the top three nations contributing to mismanaged plastics were Asian countries, namely China, Indonesia, and Vietnam [188]. Surprisingly, India, which has a similar population to China, was placed 12th but is expected to be the 5th most significant contributor to mismanaged plastic by 2025 [189].

In 2016, the region that encompasses Europe and Central Asia generated a total of 392 million tons of waste, which is equivalent to 1.18 kg of waste per person per day [190]. Even though about three quarters of this garbage has the potential to be recycled or handled through organics, recycling and composting are only recovering 31% of waste materials at the moment [187]. In addition to causing disease in the population and deteriorating air quality, this amount of waste also accelerates climate change and damages the location in which it is located [191]. Improper waste treatment procedures generated 1.6 billion tons of carbon dioxide-equivalent gasses in 2016, which accounted for 5% of global emissions [187]. On the other hand, using plastics and waste disposal lowers the living level in Thailand [192]. Landfills dispose of 15% of South Africa’s generated waste, while proper disposal methods dispose of 85% [193]. Mozambique is facing a crisis in waste management, with approximately 99% of waste remaining unprocessed and mishandled. Each year, people discard seventeen thousand tons of plastic into the ocean and river. This indicates that 10% of all plastic is in the aquatic environment [193]. The best at dealing with and controlling plastic is Menorca Island in Spain. In 2018, the nation produced 10,220 tons of plastic, 2476 tons (or 24%) of which came from the tourism industry alone. The sector in Menorca that has the most waste is tourism. Even with tourists generating 111 kg of plastic waste per capita annually, the average collection rate remains a respectable 90%. Meanwhile, 10% of Menorca’s plastic waste remains unmanaged, allowing it to decay and scatter [174]. Vietnam currently imports the majority of its plastic products. Vietnam’s accelerating industrial expansion adds more than half of the total amount of plastics to the stockpile.

The rise in plastic was a sign that the nation must manage its waste correctly. Due to the production of toxic chemicals like dioxins and particulate matter, the incineration of plastic outdoors negatively affects health and directly contributes to climate change [2,3]. By assisting the local community in protecting the beaches and coastal shorelines, both visitors and operators should assume responsibility for urging everyone to respect the environment [194]. Within the framework of its action plan for the CE [195], the European Commission has identified the issue of plastic pollution as a vital priority. However, the current method of manufacturing, utilizing, and disposing of plastics fails to capitalize on the economic benefits of a more “circular” approach and is causing considerable environmental damage [196]. Plastics are essential to modern economies, but the existing method of the processing, utilization, and disposal of plastics is not sustainable. Therefore, the environmental issues associated with plastic production, use, and consumption are the subject of increasing concern, particularly the plastic waste that accumulates annually in the oceans. The EU’s plastics strategy aims to revolutionize the design, production, use, and recycling of plastics and plastic products, with the goal of ensuring that all plastic packaging is recyclable by 2030 [197].

Single-use plastics, such as PPE kits, masks, and other items, are assisting in avoiding infections during the COVID-19 pandemic; nonetheless, there has been a significant increase in plastic pollution [198]. Ecological catastrophes brought on by the COVID-19 pandemic have previously been mentioned in several studies and reports [199]. The spread of COVID-19 brings on global health problems because protective equipment (masks and gloves) exposed to the environment threatens ecosystem services and the sustainability of the environment and harms human health and the economy [200]. Up to 40% more non-biodegradable packaging materials were consumed during the pandemic due to the increasing demand for plastic products [201]. During the COVID-19 crisis, management policies and procedures and methods for disposing of plastic trash had to be followed. Waste treatment was the intended outcome of planned waste management, which included collecting, sorting, and disposing of hospital trash on-site [202]. Despite the Biomedical Waste Management Rules of 2016, particular rules are needed to guarantee that COVID-19 wastes are disposed of scientifically. Although many nations have various institutes for research and development to address multiple issues, there are no regulatory bodies to monitor them [203]. The CE can lessen the effects of plastic pollution by lowering waste generation and identifying alternate disposal strategies. Therefore, it is necessary to consider a more robust, circular, and low-carbon economic model once more [204].

A simulation has been performed to determine the potential cost of MPs between the years 2010 and 2100 [205]. Incorporating the uncertainty in the concentration of MPs in the area and their distribution around the areas, as well as the reactions of biological impacts to either short-term or long-term exposure to MPs, has been accomplished through the utilization of simulation approaches. Because of this, it can be deduced that the cost estimates would change depending on model simulation. Because threshold values for the biological effects of MPs have not yet been determined, the data on these components of the model were produced from a best-case scenario and a worst-case scenario [205]. The best-case scenario included a response that was comparable to the one that was observed in the trials, while the worst-case scenario included essentially no reaction in shellfish.

In 2011, Constanza et al. [206] have estimated that marine ecosystem services provided society with benefits of approximately USD 49.7 trillion per year on a global scale. Most of the values used to compute this approximation were based on the maximum sustainable use (actual or hypothetical) of natural (or semi-natural) systems, which reflects functioning biomes with minimal anthropogenic disruption. A loss of USD 500–2500 billion in the value of benefits derived from marine ecosystem services is equivalent to a 1–5% decline in the delivery of marine ecosystem services annually [206].

## 7. Microplastic Waste Management

Because of the low values of all environmental loads for all selected FUs, the data suggest that thermochemical treatment might lead to a sustainable option for managing solid plastic waste [207]. From an LCA perspective, Salieri et al. [208] use worst-case scenarios to explain the importance of MPs’ discharge into freshwaters. A preliminary and condensed Characterization Factor (CF) of 3231 PAF.m^3^.d/kg was determined for the impact class of freshwater toxicity in line with the USEtox model. Two LCA case studies were carried out using this CF, the first on T-shirts fabricated from polyester and the other using shower gel that contains MPs. The findings indicate that MPs only slightly contribute to freshwater ecotoxicity in a scenario with cutting-edge wastewater treatment [208].

A sensitivity analysis of the CF and various scenarios involving the release and clearance of different MPs during wastewater treatment enabled the identification of the potential quantity of MPs contributing to environmental toxicity. Demetrious et al. [209] used LCA to assess various approaches to handling the leftovers from material recovery plants. The authors used an LCA that simulates the possible effects of acidification, climate change, eutrophication, and photochemical oxidation to assess the environmental performance of the residual waste from the material recovery plants [209]. Different waste fractions of the residual waste composition at material recovery facilities were identified using sensitivity analysis [210]. Whether credits offset power consumption or the carbon accounting techniques used to estimate biogenic carbon dioxide, the data revealed that landfills had the lowest gas emissions. Additionally, landfill emissions that were least acidifying were discovered [211]. However, the waste-to-energy (WtE) solutions were more effective in reducing photochemical oxidation and eutrophication emissions. Landfilling was found to have the lowest overall score after normalization and weighting [212].

The first stage is waste collection, which can be accomplished by source collection (by consumers) or post-separation (in centers) [213]. Source collection is recommended since it is less expensive and reduces waste contamination. Waste disposal options range from more convenient to less convenient for customers (and inversely for municipalities responsible for collection) and include Door-to-Door (D2D) waste collection with or without fees, curbside collection, and buy-back or drop-off centers (purchasing trash) [48]. Economic incentives to increase recycling rates can be positive, such as buy-back programs in which a sum of money is received (or returned) to the consumer per package or weight, or harmful, such as fees varying with weight and waste type (with lower fees for recyclables) in the D2D collection or through the use of intelligent trash containers [214]. On the other hand, setting charge values is a sensitive business: effective rates may encourage unlawful dumping or MP burning. At the same time, low fees will have little effect on waste consumption or separation [215].

Regarding avoiding MPs’ formation and accumulation, incorrect waste disposal contributes to the formation of MPs and marine litter [216]. The larger quantities of ocean litter recovered from Brazilian beaches frequented by people with lower levels of education and the rejection of microbead-containing goods by consumers exposed to awareness campaigns are examples of how effective education can be in combating MPs pollution [217]. Until recently, there was not enough knowledge about MPs’ contamination, with 73% of Chilean students unaware of the issue [218]. There is a tendency toward growing interest in this ecological issue, which is encouraged by the media and applications, free massive lectures, activities, and open online courses; beach clean-ups that are helpful for raising remediation and awareness; and cheap but effective citizen science that might aid in mapping marine waste [219].

Alternative, cutting-edge recycling technologies exist, and they can close these gaps and overcome the restrictions and constraints imposed by the plastics found in various waste streams [220]. This includes tertiary recycling solutions in which thermochemical processes convert plastic into monomers or feedstocks [221]. Other strategies for chemical recycling are also being investigated, including depolymerization, which uses chemicals to break polymer bonds, and solvent-based dissolution, which preserves polymer structure [221]. Unfortunately, it is currently unclear which recycling methods offer, in theory, the environmental advantages for each plastic use and, consequently, which methods are most appropriate for a CE.

According to their priority, the following suggested solutions are made to lessen the number of MPs that are lost to the environment during manufacture, use, and disposal [185,222,223,224,225]:

- Controlling the manufacturing and use of environmentally damaging plastic items by taxes or prohibitions while maintaining food safety and human health.
- Raising the price of virgin plastics or imposing penalties or taxes on them to increase the request for recycled plastics.
- Decreasing the utilization of plastics by eliminating useless packaging and labeling, raising awareness, and public educating, as well as by offering eco-friendly substitutes for plastics whenever possible without unintended effects.
- The introduction of waste collection methods that reduce waste generation and increase recycling rates is based on the Pay-as-You-Throw (PAYT) concept, such as deposit-refund systems and D2D waste collection.
- Recycling should be prioritized, then feedstock and WtE that enable the recovery of high-value-added products and energy, and landfill usage should be reserved only for trash created by the earlier processes.
- Decreasing and reusing waste generated through the production stage, taking ownership of waste, and mitigating the effects of products (Extended Producer Responsibility).
- Utilizing renewable energy for waste collection and recycling to lessen the negative environmental effects of recycled plastics.
- The application of LCA to enhance environmental design while taking into account the anticipated EoL of items.
- Using bio-based plastics where composting is advantageous and offering particular collection and management strategies to decrease the production of degradable plastics that produce MPs.
- Enhancing e-waste’s recycling capacity while disposing of it through waste-to-energy.

## 8. Challenges and Perspectives

Information that can be extensively integrated into LCA studies has not yet been converted from the current corpus of knowledge on MPs. At the process level, the intrinsic absence of plastic leakage data impeded the development of inventory data. Unlike other input/output fluxes, MPs that are lost or released from the value chain lack a built-in bookkeeping procedure. Therefore, the extent to which the technosphere is emitting MPs remains largely obscure. The technological and procedural constraints of the current detection and quantification methodologies further impede the accumulation of inventory data [226]. As a result of numerous research endeavors, these data gaps will ultimately be resolved. However, LCA practitioners should continue to advocate for improved methodologies, incorporate available research, and promote data transparency in this endeavor.

There are a number of significant improvements that can be introduced into LCA frameworks in order to address the growing concerns surrounding MP pollution of the environment [38]. In the beginning, the midway impact categories that are associated with human health and ecotoxicity need to be rewritten in order to take into account the physiological changes that are caused by MP particles within animals [227]. Recently, the impact categories of climate change and acidification may necessitate reconsideration, in addition to toxicity-related impact categories [228]. This is because the release of organic acids, carbon dioxide, and methane during MP mineralization can contribute to the global warming potential. It is not reasonable to suppose that all LCA studies will include a complete review of the effects of MPs from a practical point of view. In product systems where plastic leakage and losses are predicted to be limited, simple CFs may be adequate to ensure that the presence of MPs is not completely disregarded. This is the case even when MPs are not the primary focus of the study [229,230].

## 9. Conclusions

MPs are a category of threat environmental persistent pollutants that have attracted significant attention and are becoming a growing concern. The development of sustainable solutions to mitigate the adverse effects of MPs and decrease their presence in the environment is imperative as the impact of MPs continues to rise. The environmental and human health effects of MPs are quantified in existing LCA studies in this systematic review. There is a growing body of literature that illustrates the potential for MPs to contaminate the atmosphere, human food, and water sources, as well as human health through inhalation or ingestion. It is imperative that LCA practitioners play an active role in bridging the gaps between the most recent scientific discoveries and practical LCA applications in order to accurately represent the impacts of MP particles, given the current constraints. Additionally, endeavors should be undertaken to enhance the efficiency and quality of plastic alternatives, such as bioplastics, and to incorporate MP treatment technologies to reduce their negative effects and improve their removal efficiency. Ultimately, the selection of a strategy to reduce plastic use should take into account a variety of factors, including economic conditions, infrastructure, the types of MPs that are released, alternative options, and the public’s preparedness to transition to a non-plastic-dependent economy.

## Figures and Tables

**Table 1:** Ecotoxicity of PPE chemical components.

**PPE**	**Main Component**	**Degradability**	**Toxicity (g/L)**	**References**
Surgical masks	Polyurethane	Biodegradable	7.93	[11]
Medical gloves	Polyvinyl chloride	Non-biodegradable	8.8	[12]
Medical gloves	Butyronitrile	Biodegradable	33	[13]
Face masks	Polyethylene terephthalate	Biodegradable	0.8	[14]
Surgical masks	Acrylonitrile	Non-biodegradable	68.9	[15]
Surgical masks	Polyethylene	Non-biodegradable	0.13	[16]
Surgical masks	Polypropylene	Biodegradable	0.2	[17]
Surgical masks	Polystyrene	Non-biodegradable	0.3	[18]
Surgical masks	Butyronitrile	Biodegradable	33	[19]
Protective eyeglasses	Polycarbonate	Non-biodegradable	0.007	[20]

**Table 2:** Impacts of microplastics on soil microbial enzyme activities.

Soil Type	MP Type	Concentration	Impact of MPs	References
Sandy soil	PET, PA, PP, PE, PS, and PES	0.4% (*w*/*w*)	➢ MPs increased acid phosphatase activity ➢ MPs decreased β-glucosidase activity	[68]
Sandy loam soil	PA, PE, PS, PBS, PHB, and PLA	2% (*w*/*w*)	➢ Increased soil urease, phosphatase, and catalase activities	[69]
Udalfs soil	PET	0.5% (*w*/*w*)	➢ Significant decrease in the expression of methanogenesis functional genes (mcrA) and the N2O reductase gene (nosZ)	[70]
Loamy sand soil	LDPE	15 g/m^2^	➢ Increased soil β-glucosidase activity ➢ Increased soil α-glucosidase activity ➢ Increased soil leucine amino peptidase activity	[71]
Sandy loam soil	PA, PE, PS, PBS, PHB, and PLA	0.2% (*w*/*w*)	➢ Decreased soil phosphatase activity	[69]

Abbreviations: MP, microplastic; PET, polyethylene terephthalate; PA, polyamide; PP, polypropylene; PE, polyethylene; PS, polystyrene; PES, polyester; PBS, polybutylene succinate; PHB, polyhydroxy butyrate; PLA, polylactic acid; LDPE, low-density polyethylene.

**Table 3:** Impacts of microplastics on aquatic organisms.

MP Type	Organisms	Concentration	Impact of MPs	References
PHB	*Gammarus fossarum* (Koch, 1836)	100,000 particles/individual	➢ Promotes digestive constraints	[84]
PHB	*Daphnia magna* (Straus, 1820)	106.7 mg/mL	➢ Immobilization	[85]
PLA	*Clarias gariepinus* (Burchell, 1822)	0.04 g/pellet	➢ Increased counts of vibrio in the intestines	[86]
PLA	*Chlorella vulgaris* (Beijerinck 1890)	100 mg/L	➢ Stress induction ➢ Accumulation of photosynthetic pigments ➢ Increase production of antioxidant	[87]
PLA	*Mytilus galloprovincialis* (Lamarck, 1819)	80 g/L	➢ Reduce egg fertilization of larval motility ➢ Reduction in lysosome membrane stability	[88]
PHB	*Lates calcarifer* (Bloch, 1790)	0.5 µg/mL	➢ Significant necrosis ➢ Alteration in muscle ➢ Alteration in gill and heart tissues	[89]
PLA	*Danio rerio* (Hamilton, 1822)	25 mg/L	➢ Oxidative stress ➢ Depolarization ➢ Apoptosis damage of mitochondrial structures	[90]
PLA	*Artemia franciscana* (Kellogg, 1906)	1 mg/L	➢ Immobility	[91]

Abbreviations: MP, microplastic; PHB, polyhydroxy butyrate; PLA, polylactic acid.

**Table 4:** Microplastic impacts of oral exposure.

MP Type	Concentration	Exposure Route	Impact of MPs	References
PS	5 mg/kg/d	Oral gavage	➢ Intestinal damage ➢ Induction of gut microbiota dysbiosis	[126]
PE	100 mg/kg/d	Oral gavage	➢ Change in the microbial gut composition ➢ Increased levels of serum D-lactate	[127]
PS	10–100 µg/mL	Oral gavage	➢ Increased IL-6, TNF-α, and IL-1β protein expression ➢ Decreased mucosal thicknesses	[128]
PS	2 mg/kg/d	Oral gavage	➢ Chronic kidney disease ➢ Gut barrier dysfunction	[129]
PE	200 μg/g	Food	➢ Change in the microbial gut composition ➢ Increased colon and duodenum inflammation	[130]
PE	0.2 μg/g/d	Oral gavage	➢ Decreased mucus secretion ➢ Change in the microbial gut composition	[131]
PE, PS, PP, PVC, and PET	20 mg/mL	Oral gavage	➢ Histopathological damage ➢ Change in the microbial gut composition ➢ Decline in goblet cells	[132]

**Table 5:** Detection methods of microplastics.

Method	Advantages	Disadvantages	References
Raman spectroscopy	➢ Non-destructive ➢ Reliable ➢ Accurate	➢ Time-consuming ➢ Expensive instrumentation ➢ It needs sample preparation	[142,143]
FTIR	➢ Fast ➢ Non-destructive ➢ Reliable technique ➢ Accurate	➢ Be interpretable ➢ Time-consuming ➢ Expensive instrumentation	[142,144]
Transmission electron microscopy	➢ High resolution	➢ Time-consuming	[144,145]
Scanning electron microscopy	➢ High resolution	➢ Expensive ➢ Long time analysis	[146]
Pyrolysis–gas chromatography– mass spectroscopy	➢ Reduced time and cost ➢ Ideal for MPs in ➢ complex samples ➢ Reliable technique	➢ Time-consuming ➢ Destructive ➢ Lack of particle size ➢ information	[142]
Stereo microscopy	➢ Fast ➢ Easy ➢ Reliable technique	➢ Large errors ➢ Lack of small particle data	[147]
Fluorescence microscopy	➢ Immediate particle visualization ➢ Easy	➢ The ultraviolet laser can be toxic and detrimental to the sample	[144]

**Table 6:** Frequently used life cycle assessment software tools.

LCA Software	Software Platform	Key Information
Athena Impact Estimator (www.calculatelca.com)	➢ Desktop	➢ It requires a basic skill level ➢ It is mainly targeted towards architects, engineers, and researchers ➢ Data are required to be input by the user ➢ The results are shown in graphs/charts, tables, and reports
*BeCost* (http://virtual.vtt.fi/virtual/proj6/environ/ohjelmat_e.html)	➢ Web-based	➢ It presents the environmental impact of building design, construction, and maintenance ➢ It is mainly targeted towards engineers, designers, and architects ➢ The results are shown in the form of an environmental profile
Caala (www.caala.de)	➢ Web-based	➢ Mainly used for residential building applications ➢ It covers a cradle–cradle scope ➢ The results are shown in graphs/charts, tables, and reports
COCON (https://www.cocon-bim.com)	➢ Desktop	➢ This tool is primarily intended for designers who require a moderate level of expertise to work in 3D
*Eco-Quantum* (www.iva.uva.nl)	➢ Desktop	➢ Utilized for the purpose of evaluating the environmental performance of buildings and developing creative design solutions
*ECOSOFT* (www.ibo.at/en/ecosoft.html)	➢ Web-based	➢ It is mainly targeted towards architects, engineers, and researchers ➢ The results are shown in the form of three environmental indicators with an ecological index rating ranging from 0 to 100
*ELODIE* (https://info.cype.com/fr/software/elodie-by-cype/)	➢ Web-based	➢ It is mainly targeted towards architects, engineers, and consultants ➢ This tool can perform assessments for all types of buildings and neighborhoods
*Envest^®^* (https://clarityenv.com.au/envest/)	➢ Web-based	➢ It is mainly targeted towards architects, engineers, cost consultants and designers ➢ It provides an estimate of the construction cost and whole-of-life cost of the building design
*EQUER* (www.izuba.fr/logicial/equer)	➢ Desktop	➢ It is mainly targeted towards architects, designers, engineers, researchers, and consultants with moderate skills ➢ All inputs are editable at ease ➢ During the initial phases of design, this instrument can be implemented to evaluate the selection of architectural materials and form
*eToolLCD* (www.etoollcd.com)	➢ Web-based	➢ It is mainly targeted towards architects, designers, engineers, and consultants with moderate skills ➢ Users must follow a quantitative and qualitative data input format ➢ This tool covers a cradle–cradle scope
*One Click LCA* (www.oneclicklca.com)	➢ Web-based	➢ Closed-source commercial software ➢ Data inputs are allowed for editing at any stage ➢ Applicable during the early and detailed design phase
*Tally* (www.choosetally.com)	➢ Web-based	➢ Applicable during the early and detailed design phase ➢ Closed-source commercial software ➢ The results are shown in graphs/charts, tables, and reports

## Data Availability

All datasets generated for this study are included in the article.
